# Non-uremic calciphylaxis: A dermatologic complication in both MASH and alcohol-associated cirrhosis 

**DOI:** 10.5414/CNCS111578

**Published:** 2025-02-06

**Authors:** Dylan Rose Balter, Yueming Cao, James Garritano, Goran Micevic, Andrew Sanchez

**Affiliations:** 1 Yale School of Medicine,; 2 Department of Internal Medicine, Yale School of Medicine, New Haven, CT,; 3 Department of Dermatology, Baylor College of Medicine, Houston, TX,; 4 Department of Dermatology,; 5 Department of Immunobiology, Yale School of Medicine, New Haven, CT, and; 6 Department of Medicine, Beth Israel Deaconness Medical Center, Boston, MA, USA; * Equal contribution

**Keywords:** non-uremic calciphylaxis, MASH cirrhosis, Roux-en-Y gastric bypass

## Abstract

A woman with metabolic dysfunction-associated steatohepatitis (MASH) cirrhosis presented to our hospital with hepatic encephalopathy, acute kidney injury, and painful skin lesions. A skin biopsy and broad work-up led to a diagnosis of non-uremic calciphylaxis. Despite treatment with IV sodium thiosulfate therapy, the patient ultimately passed away from infectious complications. This case highlights the need to recognize non-uremic calciphylaxis, which is a dermatologic complication associated with both alcohol-associated and MASH cirrhosis. While treatment options are currently limited, recognition of non-uremic calciphylaxis is crucial for enabling honest conversations with patients about prognosis.

## Introduction 

Calciphylaxis is a life-threatening syndrome characterized by painful, non-healing skin lesions due to arteriolar calcification and thrombosis leading to skin ischemia and necrosis [1, 2]. The syndrome typically affects patients with end-stage kidney disease, when the label “uremic calciphylaxis” defines the diagnostic endpoint. “Non-uremic calciphylaxis” (NUC) refers to calciphylaxis in patients with normal kidney function or earlier stages of chronic kidney disease. While NUC is less common than uremic calciphylaxis, it carries a similarly high mortality rate: one systematic review estimated a 52% mortality rate from NUC, with the majority of deaths occurring between 2 weeks and 1 year following NUC diagnosis [3]. Alcohol-associated liver disease (ALD) is the third most common predisposing condition for NUC [2, 3]. Patients with NUC due to ALD are well described in prior reports [4, 5, 6, 7]; however, NUC patients with MASH cirrhosis are less well-described [8]. Here, we discuss the clinical course and treatment of NUC in a patient with metabolic dysfunction-associated steatohepatitis (MASH) cirrhosis. 

## Case report 

A 60-year-old female with a history of MASH cirrhosis decompensated by ascites and hepatic encephalopathy presented to the hospital after a mechanical fall related to generalized weakness and altered mental status. Her history was otherwise notable for type 2 diabetes and Roux-en-Y gastric bypass (RYGB) 15 years ago. Home medications included apixaban, lactulose, nadolol, pantoprazole, furosemide, and spironolactone. Physical examination was notable for normal vital signs, disorientation, asterixis, and multifocal duskiness over the bilateral thighs. Despite altered mentation, the patient expressed significant back and leg pain, which were initially thought to be related to her fall, as computed tomography (CT) showed a new L1 endplate compression fracture. Labs were significant for acute kidney injury (creatinine of 1.90 mg/dL from baseline 0.7 mg/dL), azotemia (BUN 24 mg/dL), normal phosphorus (3.3 mg/dL), elevated total bilirubin (2.2 mg/dL), elevated alkaline phosphatase (137 U/L), mildly elevated alanine and aspartate aminotransferases (37 and 55 U/L respectively), hypoalbuminemia (2.3 g/dL), elevated INR (1.70), and hyperammonemia (52 µmol/L). The complete blood count was unremarkable (WBC 7.3 ×1,000/μL, hemoglobin 12.6 g/dL, platelets 232 ×1,000/μL). She was diagnosed with hepatic encephalopathy due to recent lactulose dose reduction, resulting in decreased stooling. 

**Figure 1.**
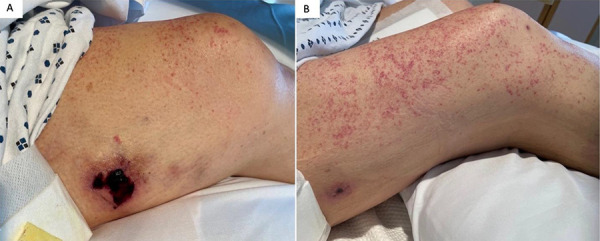
A: Left medial thigh lesion; B: right lateral thigh lesion accompanied by diffuse petechiae 11 days into admission.

**Figure 2.**
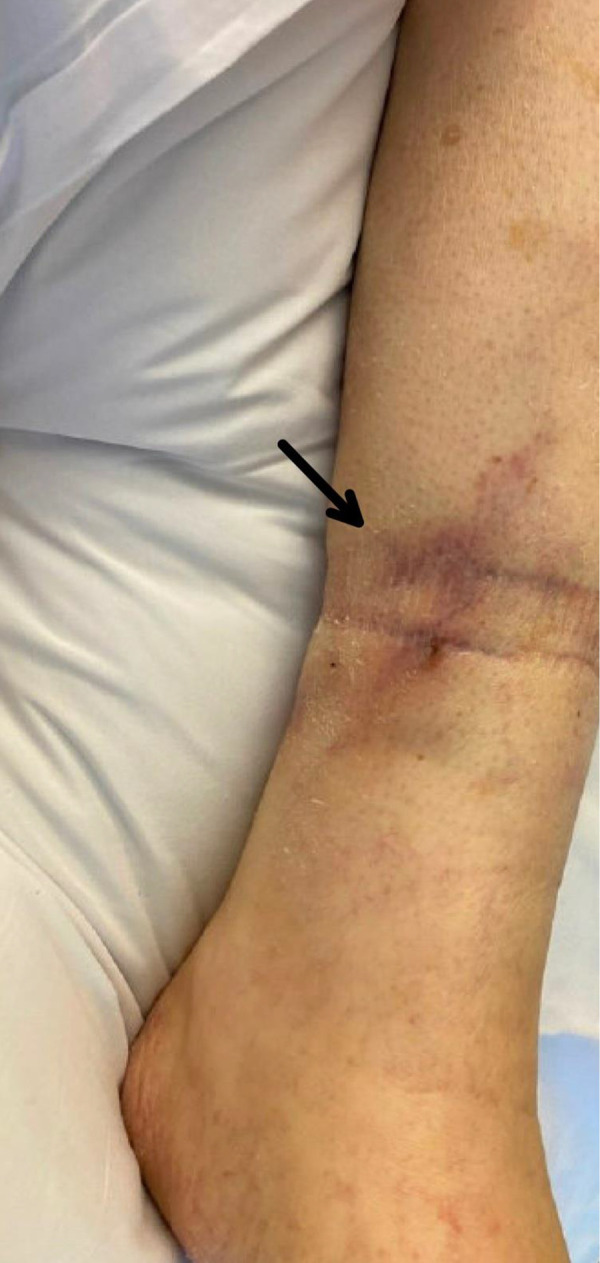
Tender retiform purpura over the left shin.

On hospital day 2, the patient reported multifocal thigh pain exquisitely tender to touch. She then developed non-palpable petechiae on all extremities despite a normal platelet count. Over several days, firm subcutaneous nodules developed over the sites of thigh pain, and retiform purpura, ulceration, and eschar formation became apparent over the right lateral thigh, left medial thigh, and right buttock (Figure 1). Tender retiform purpura were also present over the bilateral shins (Figure 2). 

**Figure 3.**
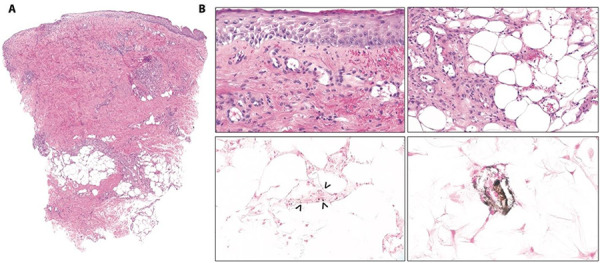
A: The skin biopsy specimen from the left medial thigh showed superficial necrosis with small vessel calcification and thrombosis. B: Top left: epidermal necrosis and hemorrhage in the papillary dermis; top right: inflammation of the subcutaneous fat; bottom: Van-Kossa stain highlights calcium deposits in small vessels (arrowheads) and subcutaneous fat.

Due to concern for calciphylaxis, biopsy of the left medial thigh eschar was performed, which showed small vessel calcification and thrombosis consistent with NUC (Figure 3). Laboratory studies revealed normal levels of parathyroid hormone (24.1 pg/mL), 25-hydroxy vitamin D (30 ng/mL), and vitamin C (0.6 mg/dL). The 1,25-dihydroxy vitamin D was elevated (115 pg/mL, reference range 26 – 95 pg/mL). Cryoglobulin, β-2 glycoprotein 1 antibody, and cardiolipin antibody testing was negative. There was low protein C activity (27%), normal protein S activity (69%), no activated protein C resistance, and low antithrombin activity (57%). Bilateral hip X-rays showed soft tissue vascular calcifications. 

The patient was started on IV sodium thiosulfate (STS) 25 g 3 times weekly on day 8 of admission. After 2 weeks of STS, 1 dose of zoledronic acid, and daily vitamin D supplementation, the patient developed septic shock secondary to wound superinfection. While she initially improved with antibiotics, hemodynamic decompensation recurred. CT of the abdomen and pelvis revealed a 7-cm abscess underlying a necrotic thigh lesion. Despite incision and drainage, 4 weeks total STS therapy, and antibiotic broadening, the patient continued to decline and passed away several days after transition to comfort care. 

## Discussion 

Calciphylaxis universally presents with painful skin lesions that are often out of proportion to exam findings early in the disease course when indurated nodules, ulceration, and eschar formation have yet to form. Duskiness suggests areas of impending skin necrosis, and retiform purpura and livedo racemosa reflect disruption of arteriolar flow [1]. Suspicion should be heightened when lesions are multifocal and located in areas rich in adipose tissue (e.g., thighs, abdomen) [2, 3]. 

The pathophysiology of NUC in patients with liver disease is unclear, but several authors cite low protein C and S levels in the context of decreased hepatic synthetic function as potential contributors [4, 5, 6, 8]. Other hypotheses include increased RANK ligand expression and decreased osteoprotegerin leading to osseous mineral loss and extraosseous calcium phosphate deposition [4]. 

Beyond MASH cirrhosis, this patient’s NUC risk factors included diabetes, female sex, and history of RYGB [2, 3]. The lattermost is a rare yet important consideration, as only 15 patients with calciphylaxis after RYGB have been reported, of which 8 had NUC [9, 10, 11, 12]. More research is required to understand whether RYGB as a risk factor may act synergistically with hepatic dysfunction. Furthermore, it is notable that our patient presented following a mechanical fall: a recent case series suggests trauma may predispose at risk patients to calciphylaxis [13]. 

The history of RYGB also increased this patient’s risk of oxalosis. Oxalosis refers to increased levels of serum calcium oxalate resulting in crystal deposition outside of the kidney, and it is categorized into primary and secondary forms. Primary hyperoxaluria comprises a group of inherited disorders, while secondary oxalosis encompasses all other etiologies that lead to increased intestinal oxalate absorption [14]. In RYGB, malabsorption allows unabsorbed fatty acids to bind to calcium, which leaves oxalate free to be absorbed into the systemic circulation [15]. Our patient presented with a necrotic thigh lesion and retiform purpura. While these two ischemic cutaneous findings have been associated with primary hyperoxaluria, the age of our patient, history devoid of recurrent nephrolithiasis, and pathology consistent with calciphylaxis was inconsistent with this group of diseases. Patients with secondary oxalosis rarely have skin manifestations but can present with calcified papules and nodules related to non-ischemic, extravascular calcium oxalate deposition [14, 16, 17, 18, 19]. 

Regarding the treatment of our patient, the terminal pathology of calciphylaxis involves arteriolar calcification as well as endothelial destruction and thrombosis [1]. In uremic calciphylaxis, calcium and phosphate derangements are frequently implicated and corrected; however, the majority of patients with NUC have normal serum calcium and phosphate levels [3]. The decision to initiate IV STS is controversial – while some reports of IV STS for NUC have shown significant recoveries, a recent meta-analysis found STS therapy for patients with uremic calciphylaxis was not associated with improved survival [2, 20, 21]. STS therapy for NUC related to liver disease has been trialed by several authors with mixed results [4, 5, 6]. The lack of effective NUC treatment options demonstrates the importance of additional research on novel therapeutic strategies. 

## Informed consent 

Verbal informed consent was obtained from the patient. 

## Authors’ contributions 

DB, YC, and JG composed the initial draft of this manuscript, which was subsequently revised by all other authors, who were additionally involved in the care of this patient. GM provided pathology images and captions. AS served as the article guarantor. 

## Funding 

None. 

## Conflict of interest 

None declared.
